# Carrier trapping and escape times in p-i-n GaInNAs MQW structures

**DOI:** 10.1186/1556-276X-9-21

**Published:** 2014-01-13

**Authors:** Hagir M Khalil, Naci Balkan

**Affiliations:** 1 School of Computer Science and Electronic Engineering, University of Essex, CO4 3SQ Colchester, UK

**Keywords:** GaInNAs/GaAs, capture rates, resonant tunnelling, p-i-n multiple quantum wells

## Abstract

We used a semi-classical model to describe carrier capture into and thermionic escape from GaInNAs/GaAs multiple quantum wells (MQWs) situated within the intrinsic region of a GaAs p-i-n junction. The results are used to explain photocurrent oscillations with applied bias observed in these structures, in terms of charge accumulation and resonance tunnelling.

## Background

Over the last couple of decades, III-V compounds containing small quantities of nitrogen (dilute nitrides) have received much attention, both experimentally and theoretically. A number of books and review articles as well as a large number of papers in the field have been published [1-3]. The interest in this material system started with the discovery of a large bowing parameter upon the addition of small amounts of nitrogen into Ga(In)As. The band gap energy is reduced with increasing nitrogen composition [4]. As a result, it has become possible to fabricate dilute nitride-based lasers, optical amplifiers and photo-detectors operating in the 1.3 and 1.55 μm windows of optical communication systems [5-7] and solar cells in multi-junction devices with increased efficiency [8,9].

In the early days of low-dimensional semiconductors, carrier capture into quantum wells of the III-V compounds was studied with considerable interest aimed at improving the performance of quantum well (QW) lasers [10]. First theoretical calculations of the carrier capture rates were performed by Shichijo [11] and Tang [12]. The mechanism was regarded as a classical process where the carrier capture rate is limited by the optical phonon scattering and the mean free path. Another calculation, presented by Burn and Bastard [13], discovered strong oscillations in electron capture rates as a function of the well width. Babiker and Ridley [14] studied the electron capture rates in GaAs QWs by taking into account the quantum mechanical aspect of the capture process with strong resonances. It has been shown that capture rates strongly depend on structural parameters such as QW and barrier widths, number of wells and the mean free path of the carriers as limited by scattering processes [13,14]. The reason for the choice of dilute nitride quantum wells is because in this study, we aimed at developing a photo-detector with a cutoff wavelength of around 1.3 μm that can be lattice matched to GaAs. Therefore, a resonant cavity-enhanced photo-detector by using GaAs/GaAlAs distributed Bragg reflectors to operate at the 1.3-μm communications window would be possible. Obviously, the main disadvantage of dilute nitrides compared to the InP-based material is the poor optical quality in devices with high nitrogen composition. This could be partly overcome by rapid thermal annealing at the expense of blue shifting of the operation wavelength.

In this paper, we present the theoretical analysis of the carrier capture and escape time in a Ga_0.96_In_0.04_ N_0.015_As_0.985_/GaAs multiple quantum wells (MQWs) situated within the built-in field of a GaAs p-i-n structure. Experimentally observed photocurrent oscillations in these structures [15,16], explained in terms of charge accumulation and field domain formation, are shown to be in accord with our theoretical results.

## Methods

### Capture time and thermionic emission

The semi-classical model used in our analysis provides useful physical insight into carrier transport across and carrier capture into the MQWs. We show that the disparity between the electron and hole capture and re-emission times from the quantum wells leads to the accumulation of electrons within the quantum wells. In our samples, the selected In and N concentrations (Ga_0.96_ In_0.04_ N_0.015_ As_0.985_) in the quantum wells ensure good lattice matching to the GaAs barriers and the substrate [10]. This allows the growth of thicker and high-quality layers and making the device suitable for photovoltaic applications where efficient absorption plays a fundamental rule [17].

In the quantum wells with the given composition, electrons are more strongly confined in the QWs (conduction band offset approximately 250 meV), than in the holes (valence band offset approximately 20 meV). The longitudinal optical (LO) phonon energy is *ħω*_LO_ *=* 38 meV [16], which is higher than the binding energy of the holes in the QW. Therefore, the holes photo-generated at the GaAs will be captured by the QW via the emission of acoustic phonons. The capture of electrons, however, will involve inelastic scattering with LO phonons which will be very fast compared to the hole capture time and assumed, in our calculations, to be negligible compared to the hole capture rates [18].

**Figure 1:**
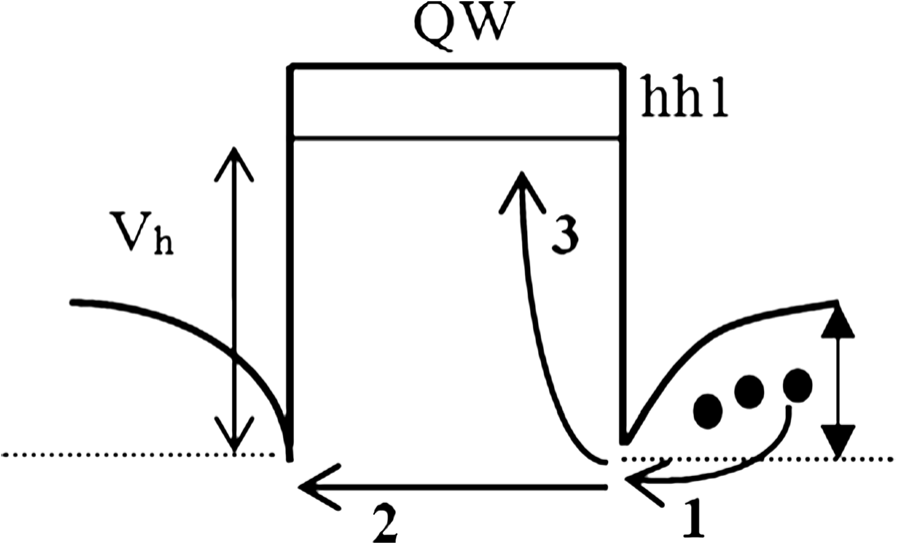
Mechanisms involved in hole capture dynamics into QW.

Under collision-free hole transport conditions, we use the following Bethe relation [19,20] to estimate the thermionic capture time for holes reaching the top of the potential barrier *Φ* (process 1 in Figure 1).

(1) $$\tau_{\text{therm}}=L_{b}\sqrt{\frac{\pi m_{h}^{*}}{2E_{h}}}\text{exp}\left(\frac{e\Phi }{k_{B}T}\right)$$

In this expression, *L*_*b*_ is the barrier width, $m_{h}^{*}$ is the heavy hole effective mass, *e* is the electronic charge, *k*_*B*_ is the Boltzman constant, and *T* is the temperature. The term *E*_*h*_ is the kinetic energy of the hole traversing the QW and can be expressed as [20,21]

(2) $$E_{h}=E_{\text{excess}}\frac{m_{e}^{*}}{m_{e}^{*}+m_{h}^{*}}+V_{h}$$

Here, *E*_excess_ is the laser excess energy, *V*_*h*_ is the depth of the QW in the valence band, and $m_{e}^{*}$ is the electron effective mass in the QW. Since the optical excitation energy above the QW band gap, the laser excess energy term is negligible.

Once the holes have reached the potential barrier edge, they can either traverse the quantum well under the influence of the built-in electric field in the p-n junction or be captured into the QW by inelastic scattering with acoustic phonons [22]. These processes are depicted in Figure 1 as processes 2 and 3, respectively. With the hole mean free path l, smaller than the QW width *L*_*w*_, the hole capture time *τ*_capture_ is reduced by the probability 1 - exp(-*L*_*w*_/*l*). The overall capture time of the hole for the GaInNAs/GaAs QW is then equal to:

(3) $$\tau_{\text{capture}}=L_{b}\sqrt{\frac{\pi m_{h}^{*}}{2E_{h}}}\text{exp}\left(\frac{e\Phi }{k_{B}T}\right){\left(1-\text{exp}\left(-\frac{L_{w}}{l}\right)\right)}^{-1}$$

In the event of not being trapped, the time for holes to traverse the QW is as follows:

(4) $$\tau_{\text{cross}}=\frac{L_{w}}{v_{d}}$$

Once the hole is captured into the well, it can escape from it via thermionic emission. The thermal escape time *τ*_*th*_ from the QW will be determined principally by the height of the barrier discontinuity and can be written as [23]

(5) $$\frac{1}{\tau_{\mathrm{th}}}=\frac{1}{L_{w}}\sqrt{\frac{k_{B}T}{2\pi m^{*}}}\text{exp}\left(\frac{-V_{h}}{k_{B}T}\right)$$

Where m^*^ is the hole effective mass in the well.

## Results and discussion

**Figure 2:**
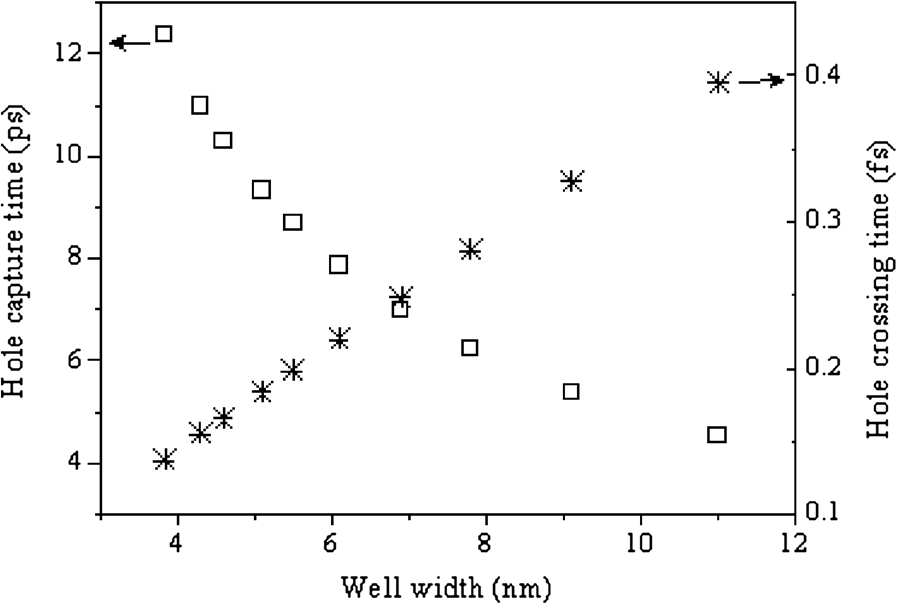
**The QW width dependence of the hole *τ***_**capture **_**(squares) and *τ***_**cross **_**(stars) calculated at room temperature.**

Using the equations above together with the band anti-crossing model [24] and the various material parameters as reported in the literature [3], the analysis of hole *τ*_capture_ and *τ*_cross_ has been carried out for the p-i-n GaInNAs/GaAs structure. The results are plotted in Figure 2 as a function of QW width.

*τ*_capture_ decreases exponentially with the QW width, as expected from Equation 3, where as *τ*_cross_ increases linearly. It is clear that the hole is more likely to traverse the quantum well than to be captured into the QW. In fact, the hole capture time is in the range of 4 to 13 ps, much longer than the 0.1 to 0.4 fs time needed to cross the QW. Thus, we assumed that at low temperatures, the last term [exp (*eΦ/k*_*B*_*T*)] in Equation 1 would be negligible.

**Figure 3:**
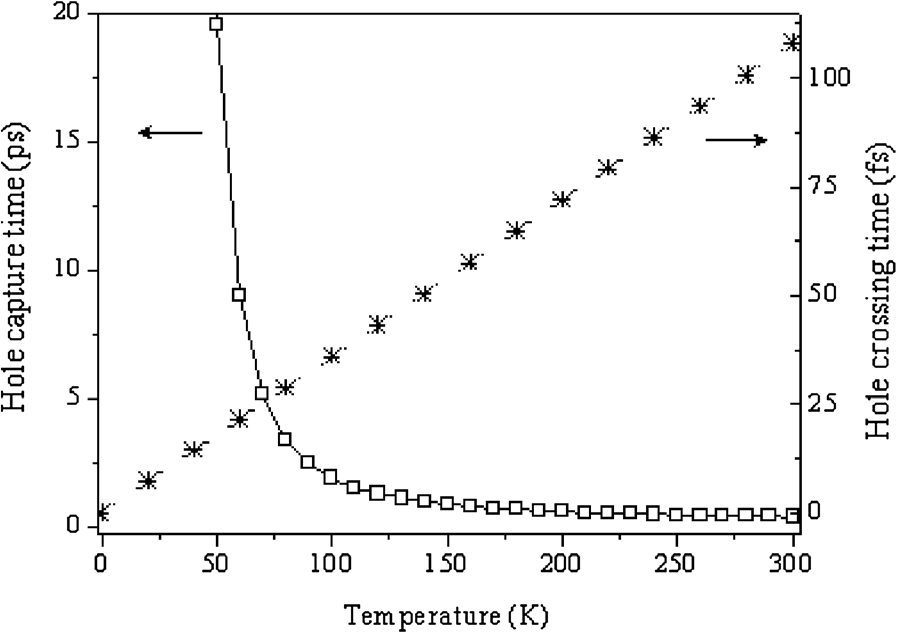
**Temperature dependence of the hole *τ***_**capture **_**(squares) and *τ***_**cross **_**(stars) calculated for a 10-nm-thick QW.**

In the current work, however, we took into account the effect of temperature and, therefore, we included this term in our calculation. The temperature dependence of *τ*_capture_ and *τ*_cross_ are plotted in Figure 3 for a 10-nm-thick quantum well.

**Figure 4:**
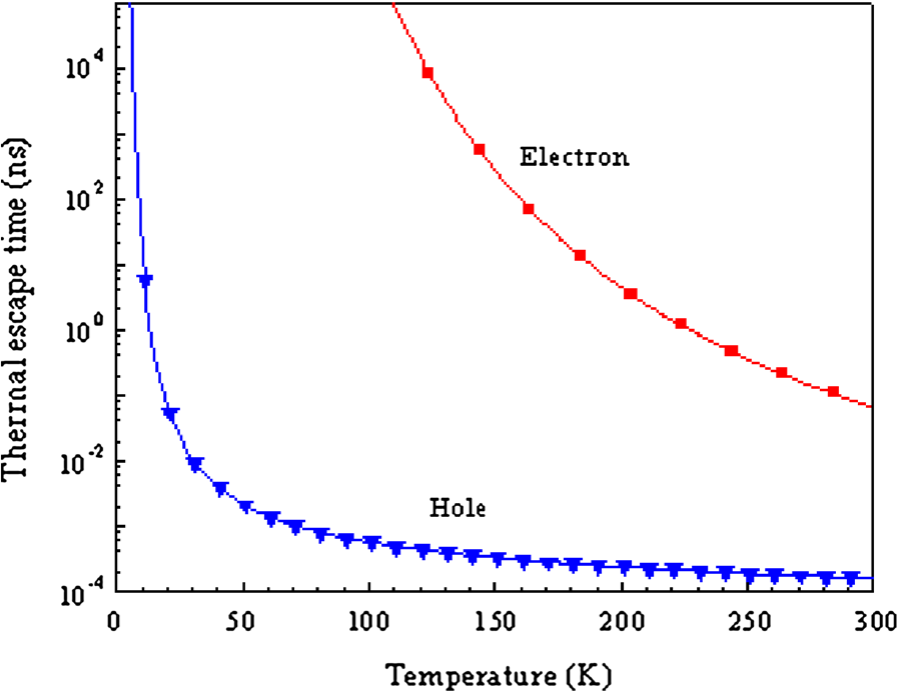
Theoretical thermal escape times for electrons and holes in the 10-nm-thick QW, as function of temperature.

The thermal escape time for both electrons and holes are also calculated as a function of temperature, using Equation 5 and plotted in Figure 4. It is clear that the hole escape time is very short, around 0.2 ps at room temperature, due to the small valence band offset. This value is two orders of magnitude shorter than the thermal escape time for electrons (approximately 60 ps). As the temperature decreases, the thermal escape time of electrons rapidly increases while for holes, the time is less than 1 ns up to temperature of *T* = 30 K, due to a lack of phonons to excite the holes over the potential barrier.

**Figure 5:**
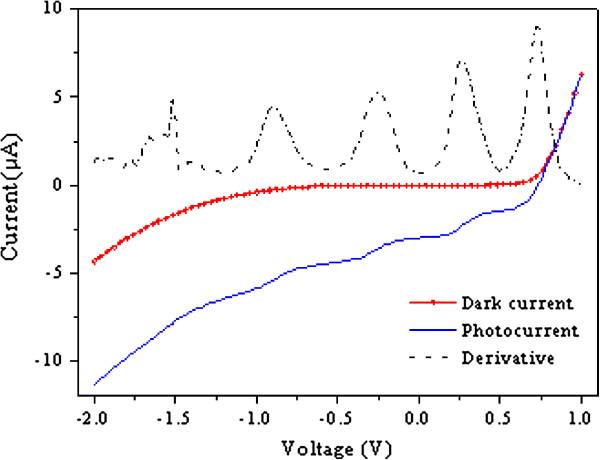
I-V results in dark and light condition, together with the derivative curves.

When the sample is under illumination with photons with energies smaller than the barrier band gap but greater than the quantum wells band gaps, photo-generated electrons will remain in the wells longer than the photo-generated holes. Therefore, accumulation of negative charge in the wells will occur. If the quantum wells are in an electric field, as they are within the built-in filed of GaAs pin structures, in our samples, thermally escaped holes will rapidly be swept away giving rise to a fast component of photocurrent. The accumulated negative charge will contribute to photocurrent via both thermionic emission and resonant tunnelling [25], giving rise to the well-known photocurrent oscillations as a function of applied voltage as shown in Figure 5, the details of which have already been reported by us elsewhere [26,27].

In Figure 5, the current is plotted against applied voltage for both in darkness and when the sample was illuminated with photons with energies greater than the quantum well band gap.

The photocurrent in Figure 5 has two components; the thermionic current which increases monotonically with applied bias and the oscillatory component which is the resonant tunnelling current [26]. In order to show clearly the oscillatory component, we took the first derivative of the photocurrent. The peak current values correspond to the resonant conditions in the wells adjacent to the anode similar to those as described in references [26,28].

## Conclusions

The aim of the work was to explain the photocurrent oscillations as a function of applied voltage that we observed in our earlier studies in GaInNAs/GaAs quantum wells placed in the intrinsic region of a GaAs pin structure. We have shown that hole thermal escape time of photo-generated holes within the quantum wells is very short compared to that of the electrons; therefore, the accumulation of negative charge in the QW may occur and give rise to the photocurrent via thermionic emission and resonant tunnelling. The resonant tunnelling component has an oscillatory behaviour with strong resonances.

## Competing interests

The authors declare that they have no competing interests.

## Authors’ contributions

HMK carried out the theoretical works, analysed the data and wrote the paper; NB supervised the project. Both authors read and approved the final manuscript.
